# Rational Design of Better Hydrogen Evolution Electrocatalysts for Water Splitting: A Review

**DOI:** 10.1002/advs.202200307

**Published:** 2022-04-18

**Authors:** Fan Liu, Chengxiang Shi, Xiaolei Guo, Zexing He, Lun Pan, Zhen‐Feng Huang, Xiangwen Zhang, Ji‐Jun Zou

**Affiliations:** ^1^ Key Laboratory for Green Chemical Technology of the Ministry of Education School of Chemical Engineering and Technology Tianjin University Tianjin 300072 China; ^2^ Collaborative Innovative Center of Chemical Science and Engineering (Tianjin) Tianjin 300072 China; ^3^ Zhejiang Institute of Tianjin University Ningbo Zhejiang 315201 China

**Keywords:** catalyst design, extrinsic effects, hydrogen evolution reaction, intrinsic effects, water‐splitting technology

## Abstract

The excessive dependence on fossil fuels contributes to the majority of CO_2_ emissions, influencing on the climate change. One promising alternative to fossil fuels is green hydrogen, which can be produced through water electrolysis from renewable electricity. However, the variety and complexity of hydrogen evolution electrocatalysts currently studied increases the difficulty in the integration of catalytic theory, catalyst design and preparation, and characterization methods. Herein, this review first highlights design principles for hydrogen evolution reaction (HER) electrocatalysts, presenting the thermodynamics, kinetics, and related electronic and structural descriptors for HER. Second, the reasonable design, preparation, mechanistic understanding, and performance enhancement of electrocatalysts are deeply discussed based on intrinsic and extrinsic effects. Third, recent advancements in the electrocatalytic water splitting technology are further discussed briefly. Finally, the challenges and perspectives of the development of highly efficient hydrogen evolution electrocatalysts for water splitting are proposed.

## Graphical abstract

This review presents varieties of representative hydrogen evolution reaction (HER) electrocatalysts benefited from intrinsic and extrinsic design strategies and gives insight into classical/novel descriptors and reaction mechanism to provide the audience with a broad and basic understanding. Moreover, the progress on water‐splitting technology is also discussed. Some invigorating perspectives on the challenges and future directions at the HER field are provided.

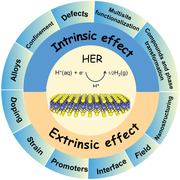

## 1 Introduction

With the intensification of the traditional energy crisis and the increasingly prominent environmental problems, changing the energy structure and increasing the proportion of renewable energy in the energy structure have become the consensus of the sustainable development of human society. The development and utilization of hydrogen energy from renewable energy has become an important development direction in the world.^[^ ^1^ ^]^ Due to its advantages of zero pollution, high efficiency, abundant sources, and wide range of uses, many countries that are plagued by environmental pollution regard hydrogen energy as the energy of the future. In particular, few impurities, zero CO_2_ emission, and environmental friendliness of water electrolysis from renewable electricity are regarded as the most promising approaches for hydrogen production.^[^ ^2^, ^3^, ^4^, ^5^ ^]^

**Figure 1:**
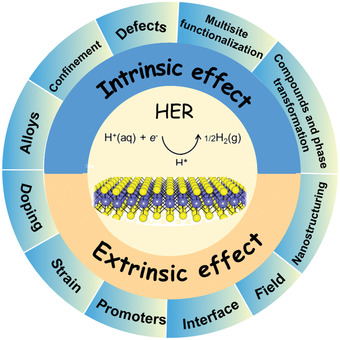
Various effects in the HER catalysts.

In the past few decades, H_2_ evolution reaction (HER), one of the half‐reactions for water splitting, has aroused considerable interest in the exploration and development of various active catalysts.^[^ ^6^, ^7^, ^8^, ^9^, ^10^, ^11^, ^12^, ^13^, ^14^ ^]^ Therefore, benefiting from detailed investigations, it is not difficult to find that catalysts with excellent performance usually have the common characteristics of high intrinsic activity, large specific surface area, and fast electron transport, which means that optimization of these characteristics should be given priority in the catalytic design and preparation process. The design principles of catalysts can cater to the above‐mentioned characteristics from the characteristics of catalysts themselves, i.e., intrinsic effects, and extrinsic assistance, i.e., extrinsic effects (**Figure** **1**). The application of the design principle of intrinsic or extrinsic effects generally does not affect the structural or electronic effect of the catalyst alone, but usually optimizes both simultaneously. For example, nanostructuring effect, smaller particles size has proven effective for enlarged the surface area (structural effect) and maximally exposed active sites (electronic effect), thus improving the electrocatalytic performance. In addition, catalysts with all key factors synergistically combined were obtained according to the catalyst design principles for further optimizing both intrinsic catalytic activity and overall catalytic performance, such as phase modulation, interfacial chemistry and heteroatomic injection defects working cooperatively to tune the catalyst electronic structure.^[^ ^15^ ^]^

Although numerous outstanding reviews relevant to HER are available,^[^ ^16^, ^17^, ^18^, ^19^, ^20^, ^21^, ^22^, ^23^, ^24^, ^25^, ^26^ ^]^ a comprehensive review which covers intrinsic and extrinsic effects, such as alloy effect, strain effect, support effect, and so on for HER is still not available. Herein, recent developments in intrinsic effect and extrinsic effect of advanced electrocatalysts including noble metals and transition metal based toward HER are summarized. First, we briefly describe the thermodynamics and kinetics of HER, as well as traditional and novel descriptors for HER. Then, the reasonable design, preparation, mechanistic understanding and performance enhancement of electrocatalysts are deeply discussed. Next, recent advancements in the proton exchange membrane (PEM) and anion exchange membrane (AEM) water splitting technologies are further summarized briefly. Finally, the challenges and perspectives of HER are proposed. We expect that this review could provide valuable guidelines and further insights into design and development of more advanced water splitting electrocatalysts.

## 2 Basic Mechanistic Principles of HER

### 2.1 Catalytic Mechanism of HER

In acid solution, HER reaction is generally considered to consist of two elementary steps. First, H^+^ near the electrode surface accepts electrons for a reduction reaction, generating hydrogen atoms adsorbed on the electrode surface (1). The adsorbed hydrogen atoms are then desorbed on the electrode surface in two ways. One is two adsorbed hydrogen atoms recombine (2), and desorbed from the electrode surface, following a Volmer‐Tafel mechanism. The other, the adsorbed hydrogen atom continues to react with another H^+^ and electron (3), according to the Volmer‐Heyrosky mechanism, so the apparent activation energy of the latter is usually higher than that of the surface recombination.

(1) $$\begin{array}{c} \mathrm{Acid}\;\mathrm{medium}:\;M+H^{+}+e^{-}\to M-H_{\mathrm{ads}}\;\mathrm{Volmer}\;\mathrm{reaction} \end{array}$$

(2) $$M-H_{\mathrm{ads}}+M-H_{\mathrm{ads}}\to H_{2}+2M\;\mathrm{Tafel}\;\mathrm{reaction}$$

(3) $$M-H_{\mathrm{ads}}+H^{+}+e^{-}\to H_{2}+M\;\mathrm{Heyrosky}\;\mathrm{reaction}$$

In alkaline solution, the HER reaction pathway is similar to that in acidic environment, but two to three orders of magnitude slower than that in acid environments and the H_ads_ intermediates of alkaline HER are formed via the water dissociation step (4).

(4) $$\begin{array}{ccc} & & \mathrm{Alkaline}\;\mathrm{medium}:\;M+H_{2}O+e^{-}\to M-H_{\mathrm{ads}}\\ & & \;+OH^{-}\;\mathrm{Volmer}\;\mathrm{reaction} \end{array}$$

(5) $$M-H_{\mathrm{ads}}+M-H_{\mathrm{ads}}\to H_{2}+2M\;\mathrm{Tafel}\;\mathrm{reaction}$$

(6) $$\begin{array}{c} M-H_{\mathrm{ads}}+H_{2}O+e^{-}\to H_{2}+M+OH^{-}\;\mathrm{Heyrosky}\;\mathrm{reaction} \end{array}$$

**Figure 2:**
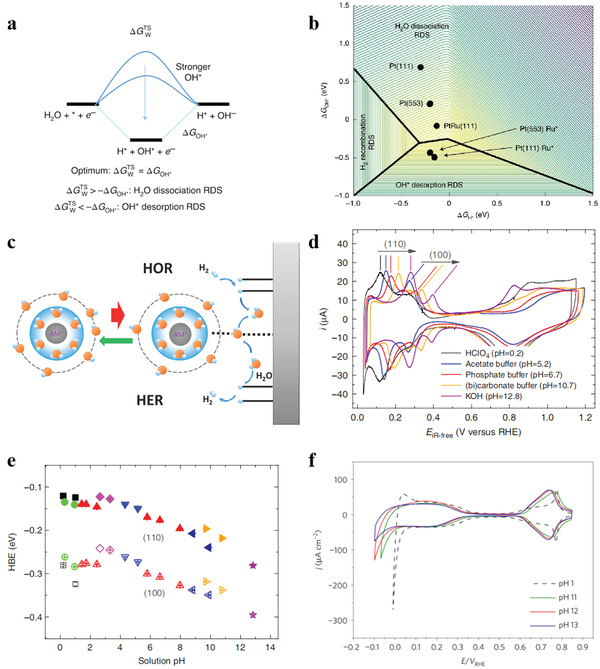
a) Schematic representation of HER reaction mechanism. b) 3D HER activity volcano. Reproduced with permission.^[^ ^34^ ^]^ Copyright 2017, Nature Publishing Group. c) Schematic representation of OH transfer mechanism in alkaline HER process. Reproduced with permission.^[^ ^35^ ^]^ Copyright 2019, American Chemical Society. d) Comparison of H_upd_ peak positions of polycrystalline Pt in electrolytes with different pH. e) Solution pH versus HBE for Pt (110) and Pt (100). Reproduced with permission.^[^ ^29^ ^]^ Copyright 2015, Nature Publishing Group. f) Comparison of H_upd_ and H_opd_ peak positions of Pt (111) in electrolytes with different pH. Reproduced with permission.^[^ ^38^ ^]^ Copyright 2017, Nature Publishing Group.

The actual reaction process is nowhere near as simple as these steps. Generally, the current theories, such as water dissociation theory,^[^ ^27^, ^28^ ^]^ hydrogen binding energy (HBE) theory,^[^ ^29^, ^30^ ^]^ and interface water and/or anion transfer theory,^[^ ^31^ ^]^ have debated the key issues in the mechanism of alkaline HER: whether water dissociation or hydrogen adsorption is the key factor to accelerate the kinetics of alkaline HER. One biggest mysteries toward alkaline HER based on water dissociation theory is, the role of OH. Recent studies have revealed that OH adsorption neither participates in the Volmer step of alkaline HER nor affects HER activity.^[^ ^32^, ^33^ ^]^ McCrum and Koper^[^ ^34^ ^]^ used the Pt decorated with Ru model to simulate electrochemical water dissociation process, demonstrating that the activation energy for water dissociation depends linearly on OH adsorption strength, even though the *OH is not a product. Combined with **Figure** **2**a,b, the reaction is bifunctional on the too‐strong binding side of the activity volcano due to the presence of *H and *OH. Conversely, hydrogen evolution is only apparently bifunctional on the too‐weak binding side of the activity volcano, even if the *OH is not necessarily a reaction intermediate, which means that activation energy of the rate‐determining step is intrinsically constrained by the *OH binding strength. Differently, Jia et al. used alkali metal cation (AM^+^) as a probe to make a thorough inquiry of the catalyst–intermediate interactions in alkaline HER. Notably, *OH is considered to be the key descriptor of the reaction, which is distinct from water dissociation theory. The kinetics in alkaline HER can be determined by catalysis of (OH_ad_)‐(H_2_O)*_x_*‐AM^+^ adducts. Apparently, increasing AM^+^ concentration can effectively promote HER. The presence of OH_ad_‐(H_2_O)*_x_*‐AM^+^ in the double‐layer region facilitates OH_ad_ desorption into the bulk, forming OH‐(H_2_O)*_x_*‐AM^+^ as per the hard‐soft acid‐base theory, thereby selectively accelerating the HER (Figure 2c).^[^ ^35^ ^]^

Despite recent discussion on the potential role of adsorbed OH for HER in alkaline, HBE theory has been shown to be the dominant descriptor for HER activity. HER in alkaline and acidic medium sharing similar reaction paths, some scholars believe that the factors determining HER activity are the same at all pH mediums and largely depends on the interaction between H and catalyst.^[^ ^36^, ^37^ ^]^ In the case of polycrystalline Pt, pH‐dependent shift of voltammetry peaks in the underpotentially deposited (UPD) hydrogen region has directly evidenced for pH‐dependent HBE (Figure 2d). As shown in Figure 2e, the HER activity of Pt (100) and Pt (110) at different pH values indicated that the pH of electrolyte affected HER activity via modifying the HBE of catalyst.^[^ ^29^ ^]^ Interestingly, similar trend does not apply to well‐defined single crystal surfaces (e.g., Pt (111)). As shown in Figure 2f, HER current and potential (*E* < 0 V) in deposition of hydrogen (H_opd_) region on Pt (111) in both acidic and alkaline medium were completely different, while they sharing similar the voltammetry curves on H_upd_ (0.05 < *E* < 0.35 V) region.^[^ ^38^ ^]^ This means that the HBE dependency theory does not seem to be universal. Meanwhile, tremendous effort has been devoted into practical materials (polycrystalline and stepped single‐crystal Pt). The H_upd_ peaks are unlikely to be independently linked to the *OH, and that the effect of oxygenated species adsorption is taken into account as well.^[^ ^39^ ^]^ In other words, the adsorbed *OH may compete with the reactive intermediate for the same surface sites and/or modify its adsorption energy, thus affecting HER kinetics.^[^ ^40^ ^]^ Besides, the electrochemical interface structure with the atomic‐scale perspective need to be taken into consideration (e.g., the potential of zero free charge (pzfc)^[^ ^38^ ^]^). Direct experimental evidences of adsorbing a small amount of Ni(OH)_2_ on Pt(111) were provided by Koper and co‐workers. They proposed that the effect of pzfc on the activation barrier of hydrogen adsorption to the energy loss associated with the reorganization of interfacial water to accommodate charge transfer through the electrochemical double layer. To be specific, the hydrogen evolution and the H_upd_ take place away from the pzfc in alkaline environment. Meaning that in alkaline environment, the interfacial water network at the HER and H_upd_ potential interacts dramatically with the strong interfacial electric field and brings about more difficult and rigid reorganization during the charge movement through the electrochemical double layer.

### 2.2 Kinetics and Thermodynamics of HER

#### 2.2.1 Thermodynamics

The HER could proceed via two pathways, namely Volmer–Tafel or Volmer–Heyrovsky reactions. Therefore, the process of converting H^+^ to H_2_ includes two successive stages: H^+^ adsorption and H_2_ generation. Generally, two corresponding computational descriptors, H adsorption energy (*E*_ad_) and the Gibbs free energy for adsorbing hydrogen atom (Δ*G*_H*_) are employed to evaluate the difficulty of the catalyst for initiating the reaction. Another parameter involved in the HER process is the Nernst potential, which reflects the thermodynamic equilibrium potential that can occur in the electrochemical reaction. In practice, HER is rarely activated at the equilibrium potential. This implies that most electrochemical processes must overcome a certain activation energy barrier. The height of the energy barrier depends largely on the nature of the interface at which the reaction takes place. Therefore, electrochemical reactions usually require more energy than thermodynamics dictates.

#### 2.2.2 Kinetics

The overpotential (*η*) is a primary parameter to evaluate the HER activity of the catalysts. The smaller the overpotential required for catalyzing the HER, the better the catalytic performance.^[^ ^41^ ^]^ The Tafel slope (*b*) is a parameter that can well reflect the inherent properties of the catalyst. A smaller b value means a lower overpotential of the catalytic process at the same apparent current density or kinetic current density.^[^ ^42^ ^]^ The exchange current density (*j*_0_) is another important parameter derived from the Tafel equation. It represents the zero potential, that is, the reaction rate under reverse equilibrium conditions. It can be understood as a descriptor of the electron transfer rate of the electrocatalyst, which determines the rate of electrochemical reaction.^[^ ^43^ ^]^ Electrochemical impedance spectroscopy (EIS) characterization is used to characterize the charge transfer properties of electrocatalysts. Charge transfer resistance (*R*_ct_) is usually used to evaluate the reaction kinetics on the electrode surface. The lower the value, the smaller the resistance of the charge movement at the interface between the electrode and the electrolyte, indicating that the electrode materials display excellent catalytic performance.^[^ ^44^ ^]^ More instructive in comparing catalysts activities and assessing their potential for large‐scale application is the activity per unit mass or conversion frequency (TOF), which is the number of reaction sites per unit of a given overpotential that catalyzes a molecule to react in a given time. If a more accurate TOF can be obtained, a clearer assessment of the intrinsic activity of the catalyst will be possible. But determining the exact number of active sites remains a big challenge.^[^ ^45^ ^]^

### 2.3 The Common Descriptors for HER

#### 2.3.1 Δ*G*_H*_

**Figure 3:**
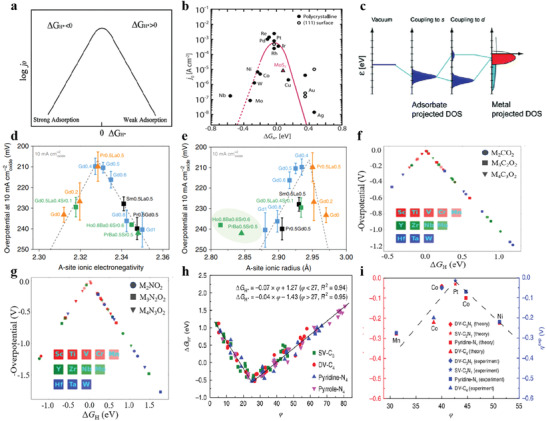
a) Relationship between *j*_0_ and Δ*G*_H*_. Reproduced with permission.^[^ ^52^ ^]^ Copyright 1958, Royal Society of Chemistry. b) Δ*G*_H*_ versus j_0_ for the catalysts. Reproduced with permission.^[^ ^53^ ^]^ Copyright 2017, American Association for the Advancement of Science. c) Schematic diagram of d ‐band center model. Reproduced with permission.^[^ ^59^ ^]^ Copyright 2005, Springer Science Business Media, Inc. d,e) A‐site ionic electronegativity and A‐site ionic radius versus overpotential for single and double perovskites. Reproduced with permission.^[^ ^64^ ^]^ Copyright 2019, Nature Publishing Group. f,g) Δ*G*_H*_ versus ‐overpotential of carbide and nitride MXenes. Reproduced with permission.^[^ ^67^ ^]^ Copyright 2017, American Chemical Society. h,i) *φ* versus Δ*G*_H*_ and overpotential on all single TM atoms supported on graphene. Reproduced with permission.^[^ ^73^ ^]^ Copyright 2018, Macmillan Publishers Ltd.

The Δ*G*_H*_ is a quantitative description for bonding strength and widely applied in the evaluation of HER activity of the catalytic sites.^[^ ^46^, ^47^, ^48^, ^49^ ^]^ According to the Sabatier principle,^[^ ^50^ ^]^ the catalytic sites should possess moderate bonding strength for the key intermediates, neither too weak nor too strong, so that the reaction intermediates can be activated and the reaction products can be easily separated.^[^ ^51^ ^]^ Pt, which is very close to the top of the volcano plot and possesses an almost thermal‐neutral Δ*G*_H*_, is known to display the best performance of HER and can achieve high reaction rates with negligible overpotential. Therefore, Pt is usually used as a measurement standard. If the catalyst is located at the left side of Pt (Δ*G*_H*_ < 0), the first Volmer step is relatively easy and H desorption is difficult, which affects the subsequent Heyrovsky or Tafel steps, eventually make the surface of the catalysts poison. On the contrary, when the catalyst stands on the right side of the Pt (Δ*G*_H*_ > 0), a relatively weak H molecule binding ability is observed. Hence, more energy is needed to start the Volmer step, which hinders the overall catalytic performance (**Figure** **3**a).^[^ ^52^ ^]^ Volcano plot can provide insight into the best catalysts for a given class of catalysts. However, as shown in Figure 3b, although MoS_2_ obtains close to the optimal Δ*G*_H*_ value, a relatively low exchange current density of MoS_2_ is observed. This is because the volcano plot is based solely on a thermodynamic descriptor, whereas other kinetic factors determine the absolute reaction rate quantitatively. Although there may be some variation in the processes involved, the active volcano plot does not move left or right, but up and down, meaning that the descriptor can still identify the bonding characteristics of the optimal HER catalyst.^[^ ^53^ ^]^

#### 2.3.2 d‐Band Center Theory

The d‐band center theory directly related H adsorption strength has been proposed as a descriptor by Nørskov for the theoretical prediction of reactants and intermediates adsorption energies on the metal and metal alloy/oxide surface.^[^ ^54^, ^55^, ^56^, ^57^ ^]^ The adsorbent H binding to the metals/alloys/oxides surface results in the strong interaction between the H electrons and the catalyst d states, thereby forming chemical bonds. Since the energy of the s‐band is essentially unchanged during bonding, the metal d orbital electrons are the main contributor to the chemical bond and determine the bond strength.^[^ ^58^ ^]^ The metal and H atoms after hybridization produce a fully filled bonding molecular orbital and a partially filled anti‐bonding molecular orbital, respectively. The bonding energy levels of the transition metals are basically the same while the anti‐bonding energy levels of different metals are quite different. These differences would induce various interactions between the metal and the H atoms and thus diverse catalytic performance. In other words, the occupancy or energy levels of antibonding orbitals ultimately affects the metal–H bond strength, which in turn influences the electrocatalytic performance. The downward/upward shift of the d‐band center increases/decreases the occupancy of anti‐bonding orbitals, reducing/increasing the antibonding state above the Fermi level and weakening/enhancing the metal–H bond strength.^[^ ^59^, ^60^, ^61^ ^]^ Therefore, an optimum d‐band center value is desired for obtaining the excellent HER electrocatalytic performance (Figure 3c).

#### 2.3.3 Valence Band Theory

If the d‐band center theory is aimed at metal atoms of active sites in the catalysts, then the valence band theory is suitable for non‐metallic carbon materials. Valence band theory chooses the difference between the highest and lowest orbital energies (*E*_diff_) of active atoms as the activity descriptor to predict the catalytic activity of nonmetallic carbon materials.^[^ ^62^, ^63^ ^]^ Here, we choose the C atoms as the active site, when the adsorbent H binds to the surface of C active center, the C 2p‐band interacts with the adsorbed H 1s‐band to form bonding and antibonding states. The increase of the filling of the bonding states due to the lower C valence orbitals may result in a stronger bonding between active C and H, thus reducing ΔG_H*_.

#### 2.3.4 Ionic Electronegativity

The A‐site ionic electronegativity (AIE) of cobalt‐based perovskites is the most predictive power for identifying a highly HER catalyst.^[^ ^64^ ^]^ In the perovskite lattice, the B site coordinated with the O anion is generally identified as the active site of HER. Theoretical calculations combined with synchrotron radiation spectroscopy reveal the potential relationship between the coordination environment (A‐site) and the active site (B–O site) of perovskite. Then, the A‐site cation could indirectly affect the catalytic activity through tuning the electronic structure and chemical state of the B site ions in the perovskite lattice. In terms of the A‐site properties, there is not only the structural parameter ionic radius, but also the thermodynamic parameter A‐O bond energy. At the same time, the electronic parameter AIE, as a unified property containing multiple structural and thermodynamic parameters, may be a good choice. Figure 3d,e reveals that the AIE value and intrinsic HER activity of perovskite exhibit the best volcano plot trend, and the performance is optimal around an AIE value of 2.33. More importantly, this design principle can also be applied to transition metal disulfide (regional electronegativity *φ*)^[^ ^65^, ^66^ ^]^ and MXenes (Figure 3f,g).^[^ ^67^ ^]^

#### 2.3.5 Generalized Coordination Number

The generalized coordination number (GCN) descriptor, considering the interaction with the next nearest neighbors and the nearest neighbors, can subtly predict the geometry of the optimal catalytic site. A set of linear proportional relationships between the binding energies of HER intermediates and GCN were established to construct a volcano‐type coordination–activity plot, from which the limiting theoretical overpotential of the catalyst could be obtained. Taking Pt as an example, the calculated GCN value of the Pt (111) is 7.5 while the GCN value of the optimal catalyst should reach 8.3 as per coordination–activity plot. The researchers designed a new Pt‐based hydrogen fuel cell catalyst with three different methods based on the above predictions. The experimental results showed that the catalytic activity could be increased 3.5 times.^[^ ^68^, ^69^ ^]^ Considering the transition metal‐based (M) single‐atom catalysts anchored on various N doped carbon substrates, M‐N_4_ center is the most frequently reported active site, such as Fe‐N_4_, Co‐N_4_ for the oxygen reduction reaction and oxygen evolution reaction, respectively, whereas the low coordination M‐N*_x_* configuration significantly promotes HER activity.^[^ ^70^ ^]^ DFT calculations reveal that the increase of N‐TM coordination number weakens the interaction between the active sites and H atom,^[^ ^71^, ^72^ ^]^ resulting in an increased Δ*G*_H*_ value. However, a relatively comprehensive and accurate descriptor (*φ*) makes it possible for rapid screening of graphene‐loaded metal single atoms catalyst and predicting HER catalytic activity:^[^ ^73^ ^]^

(7) $$\varphi \;=\theta_{d}\;*\frac{E_{M}+\alpha *\left(n_{N}*E_{N}+n_{C}*E_{C}\right)}{E_{O/H}}$$

where *E*_N_ and *E*_C_ are the electronegativity of N and C atoms respectively. *n*_N_ and *n*_C_ represent the number of N and C atoms coordinated with metal atoms, *α* is the correction coefficient, and *θ*_d_ is the number of d orbital valence electrons obtained from the periodic table of elements. The structure descriptor *φ* can be associated with Δ*G*_H*_, and can be further associated with the overpotential of HER (Figure 3h,i). A summary of some representative theoretical descriptors used for HER is given in **Table** **1**.

**Table 1:** Some representative theoretical descriptors used for HER

Descriptor	Category	Trend of catalytic activity	Applicable
Δ*G*_H*_	Electronic descriptor	Δ*G*_H*_ < 0 a relatively strong H molecule binding ability; Δ*G*_H*_ > 0 a relatively weak H molecule binding ability	All catalysts
d‐band center theory	Electronic descriptor	Downward/upward shift of the d‐band center weakening/enhancing the metal‐H bond strength	Metal and metal alloy/oxide
Valence band theory	Electronic descriptor	Increase/decrease of the filling of the bonding states enhancing/weakening the metal–H bond strength	Nonmetallic carbon materials
Ionic electronegativity regional electronegativity *φ*	Electronic descriptor	AIE—activity volcano plot Ψ—Δ*G*_H*_ volcano plot	Cobalt‐based perovskites transition metal disulfide
Generalized coordination number	Structural descriptor	GCN—activity volcano plot	Metals and metal alloys single‐atom electrocatalysts

## 3 Strategies to Improve HER Electrocatalysts

### 3.1 Intrinsic Effect

#### 3.1.1 Alloys

Constructing alloyed materials is an effective approach for the optimization and enhancement of electrochemical activities of catalysts. The main principle of alloying is to modulate the electronic structures of surface and near surface atoms of catalysts, thus modifying the adsorption/desorption strength of adsorbates on the catalysts surface.^[^ ^74^, ^75^, ^76^ ^]^

**Figure 4:**
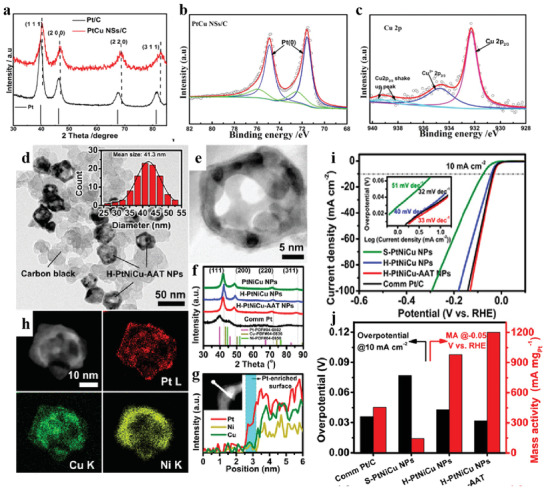
a) XRD of PtCu NSs. b,c) High‐resolution XPS spectra of Pt 4f and Co 2p. Reproduced with permission.^[^ ^83^ ^]^ Copyright 2019, Elsevier Inc. d–h) Structure of H‐PtNiCu‐AAT NPs. i,j) LSV, Tafel plots (inset), overpotential, and MA for the catalysts. Reproduced with permission.^[^ ^90^ ^]^ Copyright 2020, American Chemical Society.

Based on the Brewer‐Engel valence bond theory, the metal elements with empty or semifilled d orbitals and metal elements with more d electrons than d orbitals (i.e., the pair of d electrons) will have an electrocatalytic synergistic effect on the HER, thus greatly improving the electrocatalytic hydrogen evolution activity of the electrode. Here, we take the noble metal Pt‐based alloy as an example to discuss the alloying effect on the performance of HER. Alloying Pt with earth‐abundant metal (Fe,^[^ ^77^ ^]^ Co,^[^ ^78^, ^79^ ^]^ Ni,^[^ ^80^, ^81^ ^]^ Cu,^[^ ^82^, ^83^ ^]^ and Ti^[^ ^84^ ^]^) can significantly optimize catalytic performance of Pt‐based alloyed nanocrystals via down‐shifting the Pt d‐band center. The computed electronegativity of Pt and transition metals Cu, Ni, Fe, Ti, and Co are 2.20, 1.90, 1.91, 1.83, 1.88, and 1.30 eV, respectively, forming an electron transfer path from transition metals to Pt, resulting in the substantial negative charges on Pt surface, thus, will certainly improve the HER activity.^[^ ^85^, ^86^ ^]^ For example, Li et al.^[^ ^83^ ^]^ reported nanosingle crystal coalesced PtCu nanospheres (PtCu NSs). The resulting PtCu nanospheres display respectable HER electrocatalytic activity. The remarkable HER performance was attributed to the fact that the introduction of Cu atoms into the Pt lattice (**Figure** **4**a–c) causes the tailoring of the d‐band center and advantageous Δ*G*_H*._

In addition to the cost reduction, significant improvements in catalytic activity and structural stability demonstrated the feasibility of introducing a third transition metal into a binary counterpart. For example, PtAgCo,^[^ ^87^ ^]^ PtRhCo,^[^ ^88^ ^]^ and PtNiCo.^[^ ^89^ ^]^ For example, Wu et al.^[^ ^90^ ^]^ synthesized hollow interior and accessible surfaces of ternary PtNiCu nanostructures with low Pt‐content. The formation of hollow PtNiCu nanoparticles is attributed to the atom diffusion and galvanic replacement reaction between in situ prepared CuNi nanocrystals and Pt species. The continuous activation through pickling and annealing leads to the removal of excessive Cu and Ni from the surface, resulting in the enrichment of Pt on the surface (Figure 4d–h). Benefiting from alloy effect, hollow and open structures, the resulting catalyst displays outstanding HER catalytic properties with a small overpotential of 32 mV and a low Tafel slope of 33 mV dec^−1^ (Figure 4i,j).

#### 3.1.2 Compounds and Phase Transformation

**Figure 5:**
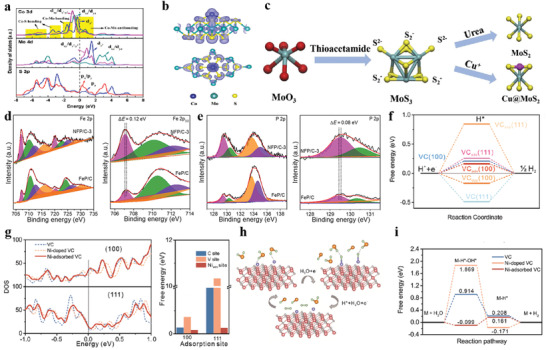
a,b) Projected densities of states and partial electron density distributions of Co‐1T‐MoS_2_ from ‐0.3 to 0.3 eV. Reproduced with permission.^[^ ^100^ ^]^ Copyright 2020, American Chemical Society. c) Mechanism of Cu atom stabilizing 1 T‐MoS_2_. Reproduced with permission.^[^ ^102^ ^]^ Copyright 2019, Elsevier B.V. d,e) High‐resolution XPS of Fe 2p and P 2p. Reproduced with permission.^[^ ^119^ ^]^ Copyright 2019, American Association for the Advancement of Science. f–i) Mechanism of Ni activation of VC. Reproduced with permission.^[^ ^133^ ^]^ Copyright 2020, Wiley‐VCH.

Transition metal compounds, such as sulfides, carbides, nitrides, and phosphides, as well as their unique phase transformation, have attracted widespread attention in the fields of HER. Some transition metal dichalcogenides (TMDs), such as MoS_2_,^[^ ^91^, ^92^, ^93^, ^94^ ^]^ ReS_2_,^[^ ^95^ ^]^ and NiS_2_,^[^ ^96^ ^]^ as an emerging 2D TMD, aroused great interest due to its unique physicochemical properties. Both experimental and DFT calculations indicate that the majority of highly active sites in transition metal sulfides are confined at the limited edges, and the bulk activity is poor. Take MoS_2_ as an example, the small size and/or abundant defects of MoS_2_ are conducive to the maximum exposure of the active edge. Besides simply exposing a large number of edge sites, attentions were also paid on engineered unsaturated sulfur edges and extremely low interplanar conductivity.^[^ ^97^ ^]^ Naturally, MoS_2_ can form three crystal structures, namely hexagonal 2H phase, rhombic hexahedron 3R phase, and tetragonal 1T phase in term of the stacking sequence and coordination of Mo atoms. For the 2H phase, the d orbital splits into d*_z_* 2, d*_x_* ^2^ *_‐y_* ^2^ *_,xy_* and d*_xz,yz_* degenerate states with an energy gap of 1 eV, while the d orbitals degenerate into d*_xz,yz,xy_* and d*_x_* 2*_‐y_* 2*_,z_* 2 of the 1T phase, and lead to the appearance of 6 electrons filling in e_2g_ orbital.^[^ ^98^ ^]^ Partial filling of orbitals provides metallic characteristics (1T) while complete filling gives rise to semiconducting characteristics (2H). HER proceeding on metallic 1 T‐MoS_2_ sustains excellent kinetics as compared to that of semiconducting 2H‐MoS_2_, because of rapid electron transfer rate and abundant catalytic active sites from basal plane and edges.^[^ ^97^, ^99^ ^]^ However, the 1 T‐MoS_2_ is a thermodynamically metastable phase and easily converts to the 2H‐MoS_2_, especially under annealing conditions. Therefore, exploring the stabilization method of 1 T‐MoS_2_ and constructing stable nanostructures will be conducive to improving the catalytic efficiency. Fortunately, this problem can be solved by introducing appropriate dopants into the 1T system. The common phase‐induced dopants are metal dopants and non‐metal dopants. Metal dopants, generally acting as electron donors, ensure a high electron density (Mo^4+^ to Mo^3+^) in the d orbital of Mo ions via their own oxidation, thus stabilizing 1 T‐MoS_2_ (**Figure** **5**a,b).^[^ ^100^, ^101^ ^]^ However, the mechanism by which copper atoms exert a stabilizing effect on 1 T‐MoS_2_ differs from the above metallic doping in that Cu is more inclined to interact with S (Figure 5c).^[^ ^102^ ^]^ The nonmetallic dopants, in this case guiding into the S vacancy, also acts as an electron donor to stabilize 1 T‐MoS_2_.

Transition metal phosphides (TMPs) have been given added attention toward HER, owing to their low electrical resistance, superior mechanical strength, and chemical stability compared with other transition metal compounds.^[^ ^103^, ^104^, ^105^, ^106^, ^107^, ^108^, ^109^ ^]^ The HER behavior of TMPs is sensitive to their structure. Many metals (such as Ni) exhibit poor hydrogen evolution activity due to the strong interaction between adsorbed hydrogen and metal surface. When phosphorus enters the crystal lattice of the metal, the presence of phosphorus can dilute the metal atoms and maintain the original electronic properties of the metal, thus lowering the free energy of hydrogen adsorption, which is conducive to hydrogen desorption.^[^ ^109^, ^110^, ^111^, ^112^, ^113^, ^114^, ^115^, ^116^, ^117^, ^118^ ^]^ Metal phosphides, distinguished from the metal sulfides, which are found active in the bulk form. Along this route, some new strategies, such as heteroatom doping, were used to boost HER catalysis. For example, Lu et al.^[^ ^119^ ^]^ prepared homogeneous hollow nanorods Ni‐doped FeP nanocrystals with carbon hybridization. XPS analyses demonstrate that the positive shift for P 2p and the negative shift for Fe 2p indicate the occurrence of the electron transfer from P atom to Fe atom, and Ni doping FeP leads to the formation of higher concentrations for electrocatalytically active phosphides, which significantly tune the electronic structure and thus high HER activity (Figure 5d,e). Pt group, Rh‐ and Ru‐based noble metal phosphide‐based^[^ ^120^, ^121^, ^122^, ^123^, ^124^ ^]^ catalysts have widely demonstrated remarkable HER activities close to or even better Pt/C. For instance, Wang et al.^[^ ^121^ ^]^ reported a new class of wrinkled ultrathin Rh_2_P nanosheets (w‐Rh_2_P NS/C) for enhancing HER catalysis. DFT calculation reveals that the P‐3p band with active open‐shell effect facilitates Rh‐4d for increased proton–electron charge exchange, which is beneficial to HER process.

Transition metal carbides (TMCs) due to their similar electronic structure as Pt and low cost, have sparked huge interest as high‐performance HER electrocatalyst.^[^ ^125^, ^126^, ^127^, ^128^, ^129^ ^]^ Nevertheless, a large number of transition metals do not occupy the d orbitals, resulting in the formation of strong M–H bonds, which hinders the rapid desorption of active hydrogen.^[^ ^130^ ^]^ Generally, doping with functional transition metal atoms and nonmetallic atoms (Fe,^[^ ^131^ ^]^ Co,^[^ ^132^ ^]^ Ni,^[^ ^133^ ^]^ N,^[^ ^134^ ^]^ P,^[^ ^135^ ^]^ and S) significantly affects the enrichment of electrons at the metal positions, resulting in a downshift of the d‐band center and weakened H‐binding toward activating TMCs. Take vanadium carbide (VC) as an instance, Ni adsorbed on VC catalyst exhibited higher HER activity and lower overpotential in alkaline medium. As shown in Figure 5f, the ∆*G*_H*_ of Ni atom adsorption on the (100) and (111) faces of VC catalyst show the lowest values, indicating that Ni atom adsorption on VC owns the best performance. The electronic states of the Ni‐adsorbed VC catalyst near the Fermi level are much higher than that of the pure VC and Ni‐doped VC catalysts, indicating excellent electrical conductivity. Furthermore, a larger density of states of the C and V on the adsorption site for (100) and (111) of the Ni‐adsorbed VC catalyst compared with those the pure VC and Ni‐doped VC catalysts at Fermi level, suggesting that both C and V sites are activated in the Ni‐adsorbed VC catalyst accompanied by prominent improvement in carrier density (Figure 5g–i).

#### 3.1.3 Defects

**Figure 6:**
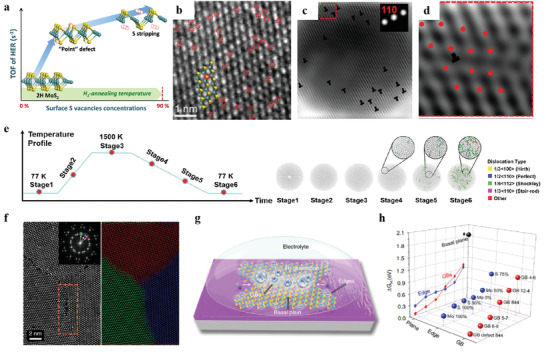
a) Evolution of the TOF of HER as a function of the surface of S concentration. b) HRTEM image of S vacancies (red cycles). Reproduced with permission.^[^ ^141^ ^]^ Copyright 2019, American Chemical Society. c,d) HRTEM images of numerous dislocations marked with T. e) Molecular dynamics simulation analysis of dislocations. Reproduced with permission.^[^ ^144^ ^]^ Copyright 2021, Wiley‐VCH. f) HAADF‐STEM image of MoS_2_ grains and GBs. g) Schematic diagram of the main types of catalytic active centers in MoS_2_. h) DFT calculations of various catalytic active centers in MoS_2._ Reproduced with permission.^[^ ^147^ ^]^ Copyright 2020, Nature Publishing Group.

Defective sites different from perfect crystals usually exhibit unique electronic properties that positively affect reactivity.^[^ ^136^, ^137^ ^]^ Therefore, defect engineering has attracted tremendous interest due to the modulation of surface charge state, the change in adsorption free energy of intermediates, the decrease of bandgap and the fact that defect sites can directly act as active sites, etc. Defects are categorized as point defects, line defects, and interface defects. Point defects (containing vacancy defects, replacement defects, interstitial defects, and antisite defects) are defined as defects that deviate from the normal arrangement of the crystal structure at a node or in a neighboring microscopic region. Vacancy defects are common point defects, such as O vacancies^[^ ^138^, ^139^ ^]^ in metal oxides and S vacancies^[^ ^140^, ^141^, ^142^, ^143^ ^]^ in 2D TMD materials. Taking the S vacancy as an example, the Δ*G*_H*_ could be modified to optimal by controlling the density of vacancies. Moreover, with the creation of S vacancies, the residual electrons confined to the S vacancies are preferable to flow to the surrounding metal atoms, leading to an electron‐rich region on the metal atoms. More precisely speaking, the delocalized electrons around metal atoms are conducive to the stronger attraction of hydrogen and promote the hydrogen adsorption on the surface, thereby adding more active sites on the inert basal plane. A representative study^[^ ^141^ ^]^ on defective MoS_2_ by Voiry and co‐workers demonstrated that HER mechanism is divided into two stages corresponding to 1) point defects at low concentrations of surface S‐vacancies and 2) undercoordinated Mo regions caused via stripping of S atoms at very high concentration of surface defects (**Figure** **6**a,b).

Linear defects (containing dislocations and steps) are periodic failures within the lattice that occur along a line near which the dislocated atoms are located. For example, Chen et al.^[^ ^144^ ^]^ induced abundant dislocations in Pt nanocrystals (Dr‐Pt) via thermal shock method at non‐equilibrium high temperature (Figure 6c,d). Dislocations caused by thermal and structural stresses in a matter of milliseconds during crystallization freeze dynamically at an ultrafast cooling rate (Figure 6e). The results show that the HER performance can be significantly boosted by introducing rich dislocations. Interface defects (containing grain boundary (GBs), twin crystals, and stratified stacking) refer to the fact that a crystal is often separated into many smaller domains by some interfaces, which have higher atomic arrangement integrity and more serious atom misalignment near the interfaces between the domains. GBs are common interfacial defects in atomically thin or 2D polycrystalline materials. The presence of GBs has been recently shown to boost the catalytic activity in metals.^[^ ^145^, ^146^ ^]^ For example, He et al.^[^ ^147^ ^]^ designed nanocrystalline thin films with ultrahigh densities GBs in wafer size (Figure 6f). The resulting nanograin film exhibited a superior H_2_‐evolution performance in acid solution, which can be attributed to the activation of sites on the otherwise HER‐inert basal plane of the MoS_2_ by the presence of GBs (Figure 6g,h).

#### 3.1.4 Confinement

**Figure 7:**
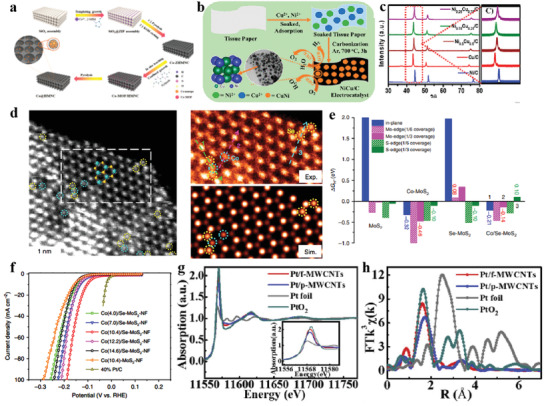
a) Schematic representation of the synthesis of Co@HMNC. Reproduced with permission.^[^ ^148^ ^]^ Copyright 2021, Wiley‐VCH. b) Synthetic strategy for NiCu/C. c) XRD of NiCu/C. Reproduced with permission.^[^ ^153^ ^]^ Copyright 2020, American Chemical Society. d) Structural characteristics of Co/Se co‐doped MoS_2_ nanofoam (Co/Se‐MoS_2_‐NF). e) DFT calculations of Co/Se‐MoS_2_‐NF. f) LSV for the catalysts. Reproduced with permission.^[^ ^154^ ^]^ Copyright 2020, Nature Publishing Group. g,h) XAFS of the Pt/f‐MWCNTs. Reproduced with permission.^[^ ^161^ ^]^ Copyright 2019, Elsevier Ltd.

The confinement effect refers to the phenomenon that the abnormal distribution of local electron cloud and the relatively closed structure of reaction region restrict the molecular movement and promote some catalytic reactions. Confinement effects are generally divided into three categories: spatial‐confined, lattice‐confined and cover‐confined. Spatial‐confined often involves the employment of mesoporous carbon or metal‐organic frameworks (MOF), whose micropores effectively control the particle size of the catalyst. A good such example was reported by Wang and co‐workers, who succeeded in preparing a breathable electrocatalyst (denoted as Co@HMNC) based on N‐doped graphitic carbon with Co nanoparticles spatially confined in an inherited honeycomb‐like macroporous structure (**Figure** **7**a).^[^ ^148^ ^]^ The obtained Co nanoparticles (a diameter of 10–11 nm) were uniformly embedded in carbonous macropores by means of “MOF‐in situ‐leaching and confined‐growth‐MOF” strategy. The acceleration of triple transport and fast reaction kinetics are largely attributed to the “ships in a bottle” spatial confinement effect. Moreover, the small size and high dispersion of noble metal particle/clusters confined to MOFs^[^ ^149^ ^]^ lead to a significant reduction in the loading capacity of noble metals to HER.

The cover‐confined metals refer to the metal^[^ ^150^ ^]^ or metal alloy nanoparticles,^[^ ^151^, ^152^ ^]^ encapsulated in materials. As for carbon materials such as graphene and carbon nanotubes, due to their stable and chemically inert, they tend to provide active metal catalysts with effective and tight protection from the harsh conditions. As a result, this type of catalyst offers great possibilities for the use of reactive but perishable non‐noble metals under extreme conditions. Moreover, the difference in work function causes electrons to transfer from the metal to the cover, which will optimize the electron state or work function on the outer surface of the cover, triggering their catalytic activity and thus activating the reaction. For example, Ahsan et al.^[^ ^153^ ^]^ developed a facile synthesis of bimetallic nickel–copper (NiCu) alloy nanoparticles confined in a sp^2^ carbon framework (Figure 7b,c). STEM images confirmed the incorporation of Ni_0.25_Cu_0.75_ nanoparticles into the porous carbon structure. Benefiting from desirable structure, the Ni_0.25_Cu_0.75_/C displayed pronounced HER performance and considerable electrochemical stability.

The lattice‐confined single atoms are defined as the single atoms doped or anchored in the coordination environment of the catalyst lattice (host matrix). Meanwhile, aggregation of active species can be effectively mitigated through confining single atoms to the host matrix lattice. Furthermore, the electronic states generated by the interaction of single atoms and host matrix may enhance the electrocatalytic activity and kinetics of materials.^[^ ^154^, ^155^, ^156^ ^]^ For example, Zheng et al.^[^ ^154^ ^]^ synthesized a MoS_2_ nanofoam catalyst co‐confining Co in inner layer and Se in surface (denoted as Co/Se‐MoS_2_‐NFs). Atomic‐resolution HAADF‐STEM images validate that Co and Se atoms are embedded around Mo and S atoms and Co and Se atoms present adjacent or separate distributions (Figure 7d). DFT calculations reveal that inner layer‐confined Co atoms encourage surrounding S atoms while surface‐confined Se atoms stabilize the structure and the synergistic effect results in abundant active sites generated on the inner and edge of the plane with moderate hydrogen adsorption performance (Figure 7e). The Co/Se‐MoS_2_‐NFs manifests outstanding HER catalytic performance with a small overpotential of 188 mV at 100 mA cm^−2^ (Figure 7f).

#### 3.1.5 Multisite Functionalization

The concept of functionalization effect originated from organic chemistry. Now this concept is widely used in the field of catalysis. The steric and electronic effects of the original or new groups formed after the reaction, as well as the electrostatic effect of electrons, may affect the capture reactants, intermediate products, and electronic structures of the adjacent active sites, thus affecting the catalytic activity. Carbon materials have been extensively investigated because of their good electrical conductivity and the well‐developed pore structure, which is conducive to mass transfer. Nevertheless, carbon materials generally cannot provide effective anchoring for direct coupling of other functional metal‐based materials due to the high degree of graphitization. It is generally believed that modifying the substrates with functional groups enhanced the electrocatalytic activity of the composites while simultaneously obtain more exposed active sites.^[^ ^157^, ^158^, ^159^ ^]^ Common functional groups are halogen atoms,^[^ ^160^ ^]^ alcohols, aldehydes, carboxylic acids, and so on. Investigation on the impact of functionalization (multiwall carbon nanotubes) for Pt single‐atom and cluster catalyst (denoted as Pt/f‐MWCNTs) prepared via photodeposition possessed a higher mass activity of 18.16 A mg_Pt_^−1^@‐0.05V.^[^ ^161^ ^] ^X‐ray absorption spectroscopy characterizations indicate that a Pt atom in f‐MWCNTs supporter was probably coordinated with four O atoms of OOH (Figure 7g,h). The high‐density unoccupied d‐orbitals of Pt in Pt/f‐MWCNTs have proved to be beneficial for the combination with H 1s orbital. Furthermore, OOH with negatively charged around the Pt sites is capable of decreasing the H^+^ concentration polarization, thereby improving the activity and stability.

### 3.2 Extrinsic Effect

#### 3.2.1 Strain Effects

Tuning surface strain has been proven as an efficient strategy for regulating the surface electronic properties and then the catalytic performance of metal catalysts.^[^ ^162^, ^163^, ^164^, ^165^, ^166^, ^167^, ^168^, ^169^, ^170^ ^]^ The strain effect on the atomic scale can lead to the change of lattice parameters, change the inherent atomic distance and modify the energy level of bonding electrons, thus greatly reducing the energy level barrier of HER.

**Figure 8:**
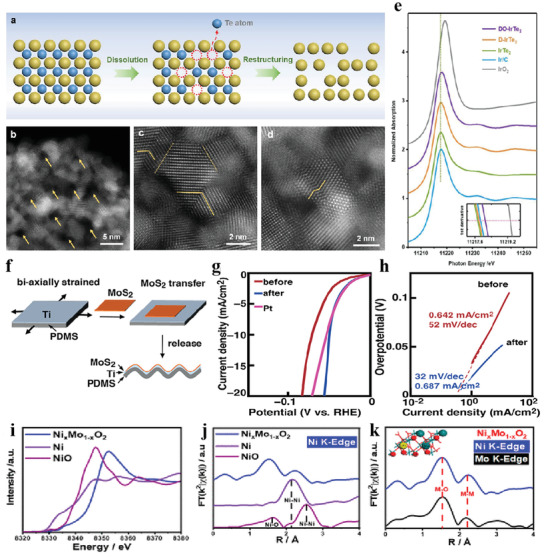
a) Schematic diagram of defect‐rich structure formation. b–d) Atomic‐resolution HAADF‐STEM images of D‐IrTe_2_. e) XANES spectra of D‐IrTe_2_ HNSs. Reproduced with permission.^[^ ^177^ ^]^ Copyright 2020, Wiley‐VCH. f) Schematic diagram of the crumpling process of MoS_2_ film. g, h) LSV and Tafel plots of the film before and after the crumpling. Reproduced with permission.^[^ ^182^ ^]^ Copyright 2020, American Chemical Society. i–k) XAFs spectra of Ni*_x_* Mo_1‐_ *_x_* O_2._ Reproduced with permission.^[^ ^199^ ^]^ Copyright 2020, American Chemical Society.

Lattice mismatch generally refers to the phenomenon caused by the inconsistency of lattice constants between two kinds of crystals. The common synthesis processes for lattice mismatches are dealloying^[^ ^171^, ^172^, ^173^ ^]^ and epitaxial growth.^[^ ^174^, ^175^, ^176^ ^]^ Dealloying, also known as selective corrosion, is the selective removal of one or more active components (sometimes called base components) via chemical or electrochemical methods, depending on the differences in chemical activity between the different components in the alloy. The remaining components (also referred to as noble components) spontaneously form 3D discontinuous porous metals by atomic diffusion and aggregation. Dealloying can produce strain and other effects that reduce the loading of noble metal catalysts and/or improve HER catalytic performance. For example, Pi et al.^[^ ^177^ ^]^ found that the structure of IrTe_2_ hollow nanoshuttles (HNSs) strongly relied on the potential applied in the electrochemical dealloying, while the mild potential result in the formation of IrTe_2_ HNSs with metal Ir shell (D‐IrTe_2_ HNSs) (**Figure** **8**a). Such electrochemical dealloying is beneficial to the generation of defects (holes, twins, and grain boundaries), resulting in the increase in the lattice strain of the reconstructed IrTe_2_ HNSs, which are illustrated in Figure 8b–e. Moreover, D‐IrTe_2_ HNSs display splendid catalytic performance with low overpotential of 54 mV at 10 mA cm^−2^ in alkaline solution. Heterogeneous epitaxy has long been applied in the preparation of functional heterostructures or junctions via vapor deposition. WS_2_^[^ ^176^ ^]^ confined epitaxy grows in ordered mesoporous graphene characterized by nanocrystalline superlattice, and the spherical curvature exerted by the mesoporous graphite makes the growing WS_2_ nanosheet produce strain and S‐vacancy uniformly. DFT calculations reveal that strain plays a key role in enhancing HER activity.

2D materials have inherent advantages (flexible, strong, and sensitive to strain) in utilizing strain effects for modulation of their intrinsic physical or chemical properties. Therefore, both theoretically^[^ ^178^, ^179^ ^]^ and experimentally^[^ ^180^ ^]^ have conducted intensive investigations for the impact of strain on the electronic structure for 2D materials. For simplicity, these works are divided into two categories: strained on flexible substrate and that on hard substrate depending on the source of strain. For example, Xiong et al.^[^ ^181^ ^]^ proposed that the surface strain stemming from rigid carbon microflower scaffold (surface)–MoS_2_ nanosheets (substrate) effectively alleviates agglomeration (nanosheets), which contributes to boosted activity. Compared with rigid substrates, flexible substrates not only act as templates but also provides potential applications of industrial field, even extreme environments. Li et al.^[^ ^182^ ^]^ reported the effect of Ti‐coated flexible polymer substrates on electrocatalytic HER of defective MoS_2_ based on experimental investigation and DFT calculations. Figure 8f shows that a monolayer MoS_2_ film was transferred to the top of the Ti layer which was biaxially deposited on a prestrained polymer substrate with a diameter of 100 nm, and then release the strain of the polymer substrate. It is demonstrated that the modification of chemical nature and forming an interfacial tunneling barrier account for the observed improvement in activity after releasing the strain of the polymer substrate (Figure 8g,h).

#### 3.2.2 Doping

Heteroatom doping directly and continuously fine‐tunes electronic structure and HER activity of the catalyst without changing the chemical composition.^[^ ^183^, ^184^, ^185^, ^186^, ^187^, ^188^, ^189^, ^190^, ^191^, ^192^, ^193^, ^194^, ^195^ ^]^ Therefore, heteroatom doping is an effective strategy widely used to optimize HER activity. Transition metal doping (Fe,^[^ ^196^ ^]^ Co,^[^ ^197^, ^198^ ^]^ Ni,^[^ ^199^ ^]^ Zn,^[^ ^200^ ^]^ and Mo^[^ ^201^ ^]^) modifies the local electronic environment and can also optimize the interatomic distances and coordination numbers of active sites. The ligand and strain effects produced by the introduction of transition metals can adjust the Δ*G*_H*_. For example, metal Ni atom doped MoO_2_^[^ ^199^ ^]^ displays excellent HER catalytic performance and robust stability in acidic environment. The XANES and EXAFS characterizations reveal that Ni atom is doped via substituting Mo atoms in MoO_2_ (Figure 8i–k). The electron deficiency of the neighboring O sites caused by Ni doping leads to the adsorption of H on O sites, thus increasing the coverage of surface H.

In addition to metal doping, non‐metal doping also exhibits distinctive features and superior HER activity. Unlike metal‐doped strategy, non‐metal doping cannot only modify the Δ*G*_H*_ or the energy level matching degree but also lead to amorphous structure or crystal distortion with plenty of active sites. Recently, many researchers focused on regulating HER kinetics and enhancing their electrocatalytic activities by incorporating nonmetal elements (N,^[^ ^202^ ^]^ O,^[^ ^203^ ^]^ S,^[^ ^204^, ^205^, ^206^ ^]^ and P^[^ ^207^ ^]^) in the transition metal‐based HER electrocatalysts. For example, Xue et al.^[^ ^206^ ^]^ fabricated S atom doped into CoSe_2_ nanosheets by a solvothermal ion‐exchange approach. DFT calculations unambiguously validate that S doping drives ΔG_H*_ close to the optimal value and remarkably decreases the kinetic barrier energy of Heyrovsky reaction for S‐CoSe_2_‐catalyzed HER, thus increasing the HER performance. Non‐metal doping, especially co‐ and/or tridoping, can effectively tune the electronic properties of the neighboring atoms and thereby boost their electrocatalytic performances. Liu et al.^[^ ^203^ ^]^ fabricated P and O dual‐doped MoS_2_ nanosheets. Particularly, the P–O bonds formed by P and O dual‐doped link MoS_2_ nanosheets into a continuously integrated framework to achieve rapid electron transfer. Likewise, the as‐generated P‐O bonds can prevent further agglomeration and restacking of MoS_2_, thereby maximizing the exposed active sites density.

#### 3.2.3 Promoters

The promoter effect is the change of catalyst performance (physical and chemical factors) caused by the addition of promoters. As is well known, Pt‐based materials due to their excellent electronic structures are the most active and advanced catalysts toward HER in both acid and alkaline solutions. However, a slower kinetics exists in the Volmer reaction of alkaline HER mainly because of the lack of an optimal active site for cleaving H–O–H covalent bond. Therefore, the catalytic activity of Pt in alkaline medium is two to three orders of magnitude weaker than that in acid medium. Henceforth, a strategy realizing a catalyst with one component as water dissociation promoter and the other one for H^+^ reduction and H_2_ formation was adopted to boost the alkaline HER activity.^[^ ^208^, ^209^, ^210^, ^211^, ^212^, ^213^, ^214^, ^215^ ^]^ However, H^+^ is easily obtained from aqueous solutions in acidic media. Therefore, promoters often play other vital roles in the process of electrocatalysis.^[^ ^216^, ^217^, ^218^, ^219^, ^220^ ^]^ For example, Ti_3_C_2_ MXenes^[^ ^216^ ^]^ and N‐doped graphdiyne (NGDY)^[^ ^217^ ^]^ behave as promoters to accelerate charger transfer, achieving highly efficient HER electrocatalysis. In addition, promoters could also facilitate the formation of catalytic active sites. For example, Kuraganti et al.^[^ ^218^ ^]^ proposed that employment substitution dopants activate MoSe_2_ and TMD‐like activity by promoting the formation of active Se‐vacancies, rather than enhanced their inherent activity based on experiments and DFT calculations. Besides, some promoters serve to optimize the electronic properties of the active centers, thereby enhancing catalytic activity. Liu et al.^[^ ^219^ ^]^ reported heterogeneous nanowires containing MoO_2_ and Ni nanoparticles, which are encapsulated in N‐doped carbon layers. DFT calculations reveal that the N‐doped carbon layer behaves as an active adsorption site for hydrogen. The internal MoO_2_‐Ni species act as effective promoters to coordinately optimize the hydrogen adsorption/desorption strength on the interface carbon, making the active sites more efficient.

#### 3.2.4 Nanostructuring

**Figure 9:**
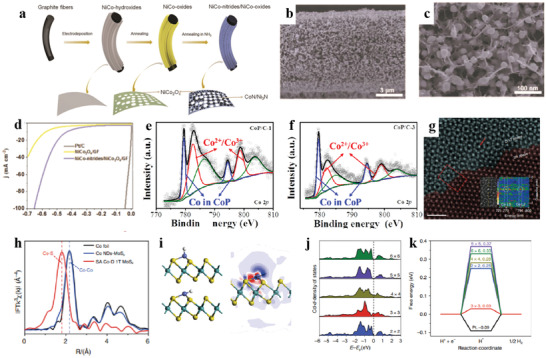
a) Synthetic strategy for NiCo‐nitride/NiCo_2_O_4_ supported graphite fibers. b,c) SEM images of NiCo‐nitrides/NiCo_2_O_4_/GF. d) LSV of the catalysts. Reproduced under the terms of the Creative Commons CC‐BY license.^[^ ^248^ ^]^ Copyright 2019, The Authors. Published by Wiley‐VCH. e,f) High‐resolution XPS spectra of Co 2p. Reproduced with permission.^[^ ^249^ ^]^ Copyright 2019, Elsevier. g–k) HAADF‐STEM image, XANES, and DFT calculations of SA Co‐D 1T MoS_2._ Reproduced with permission.^[^ ^276^ ^]^ Copyright 2019, Nature Publishing Group.

Nanomaterials as catalytic materials have important application value in energy conversion and storage devices.^[^ ^221^, ^222^, ^223^, ^224^, ^225^, ^226^, ^227^, ^228^, ^229^, ^230^, ^231^, ^232^, ^233^, ^234^, ^235^ ^]^ There are many classification methods of nanomaterials, according to their structures can be divided into: 0D,^[^ ^236^, ^237^, ^238^, ^239^ ^]^ 1D,^[^ ^240^, ^241^, ^242^ ^]^ 2D,^[^ ^243^, ^244^ ^]^ and 3D nanomaterials.^[^ ^245^, ^246^, ^247^ ^]^ For example, Liu et al.^[^ ^248^ ^]^ reported the 3D niconitrides nanoparticles/NiCo_2_O_4_ nanoflakes/graphite fibers (NiCo‐nitrides/NiCo_2_O_4_/GF), which was designed and fabricated by assembling nitride‐oxides hetero‐nanostructures onto graphite fibers via a simple electrochemical deposition and subsequent in situ nitridation (**Figure** **9**a). The target catalyst possessed uniformly distributed nanospheres with a diameter of 30–50 nm, which are assembled on the surface of the interconnected nanoflakes to form a unique 3D beaded fabric‐like nanostructure (Figure 9b,c). Impressively, NiCo‐nitrides/NiCo_2_O_4_/GF display a relatively low HER overpotential of 71 mV for 10 mA cm^−2^ in 0.1 m KOH (Figure 9d). The highly improved alkaline HER performance arises from the unique 3D structures achieved by synthetic strategy mentioned above.

The size effect plays a significant role in the nanostructuring strategy of catalysts. The smaller crystal size if well dispersed increases the utilization of the active material, thus improving the catalytic activity. The size of the catalyst is limited while maintaining the reactivity and avoiding aggregation to maximize the reactivity. Size effects could also induce some intriguing phenomena. For example, when the grain size is small to a certain value, the electron level near the metal Fermi level changes from quasi‐continuous to discrete level, resulting in the existence of discontinuous highest occupied molecular orbitals (HOMO) and lowest unoccupied molecular orbitals (LUMO), leading to a wider energy gap and an increased surface energy. The decrease of grain size causes the change of atom transport and electron energy spectrum on the surface of nanocrystals, which results in the high catalytic activity of the surface atoms. However, a recent study found that 9.2 nm CoP (CoP/C‐3) particles display higher intrinsic HER‐activity performance than 3.3 nm CoP (CoP/C‐1) particles, which is attributed to the severe oxidation of smaller CoP particles once exposed to air. XPS analyses reveal that most of the cobalt in 9.2 nm CoP (CoP/C‐3) particles remains in a quasimetallic state (Figure 9e,f), which is conducive to the desorption of H_ads_.^[^ ^249^ ^]^

#### 3.2.5 Interface

Interface effect refers to the interface between two types of active materials due to strong bonding, electronic interaction or synergistic effect, which can form more active centers than individual components.^[^ ^250^, ^251^, ^252^, ^253^, ^254^, ^255^, ^256^, ^257^, ^258^, ^259^, ^260^, ^261^, ^262^, ^263^, ^264^, ^265^, ^266^, ^267^, ^268^, ^269^ ^]^ The complex electronic structures of interfacial electrocatalysts make the fundamental investigations of structure‐activity relationships still remain as a great challenge. Nevertheless, this complexity also introduces various structural features that can be adjusted advantageously. For example, reasonable design and construction of active materials with high energy interface structure. It has been recently proved that interface engineering, including MoS_2_/CoS_2_,^[^ ^9^ ^]^ Mo_2_CT*_x_*/2H‐MoS_2_,^[^ ^270^ ^]^ Ru‐Mo_2_N,^[^ ^271^ ^]^ Co_9_S_8_‐MoS_2_/NF,^[^ ^272^ ^]^ Mo_2_C/MoSe_2,_^[^ ^273^ ^]^ CoP‐MoO_2_/MF,^[^ ^274^, ^275^ ^]^ etc. could modify the electronic structure at the interface sites for reducing the kinetic energy barrier for the hydrogen generation pathway. For example, Zheng et al.^[^^]^ successfully synthesized an interface catalyst covalently bonded with distorted 1T‐MoS_2_ nanosheets (SA Co‐D 1T MoS_2_) by atomic Co arrays. HAADF‐STEM images confirm that the atomically isolated Co species dispersed on the D‐1T MoS_2_ matrix and the obvious interface between 2H MoS_2_ and SA Co‐D‐1T MoS_2_ (Figure 9g). DFT calculations reveal that the synergy between the Co adatom and S of the D‐1T MoS_2_ support produces an ensemble effect to optimize the H‐binding mode on the interface, thus obtaining superior catalytic behavior (Figure 9h–k).

#### 3.2.6 Field Effect

Field‐assisted electrocatalysis is an electrocatalysis reaction with the assistance of an applied electric field and/or magnetic field. It represents a promising approach because it provides more freedom in the design of catalysts and the process of electrochemical reactions.

**Figure 10:**
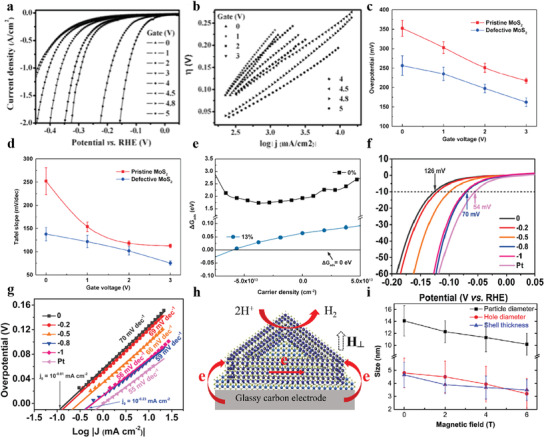
a,b) LSV and Tafel plots at different back gate voltages. Reproduced with permission.^[^ ^277^ ^]^ Copyright 2016, Wiley‐VCH. c,d) Overpotential and Tafel slope as a function of gate voltage. e) The variation for the ΔG_H*_ of pristine MoS_2_ (black) and defective MoS_2_ (blue) with carrier density. Reproduced with permission.^[^ ^278^ ^]^ Copyright 2019, American Chemical Society. f,g) LSV and Tafel plots at different back gate voltages. Reproduced with permission.^[^ ^279^ ^]^ Copyright 2017, American Chemical Society. h) Schematic illustration of electron transfer. Reproduced with permission.^[^ ^285^ ^]^ Copyright 2020, American Chemical Society. i) Magnetic field versus size plot. Reproduced with permission.^[^ ^288^ ^]^ Copyright 2019, Elsevier Ltd.

More recently, external electric fields have been applied to the electrocatalytic process to enhance HER activity, thus affecting the reaction pathway and producing a net positive effect. As is well known, the electrical conductivity of catalysts has a strong influence on their activity. Taking MoS_2_ as an example, researchers have recently revealed that their catalytic activities are sensitive to an external electric field. With an additional gate voltage of 5 V,^[^^]^ the sample further decreases the overpotential and Tafel slope from 240 to 38 mV and 200 to 110 mV dec^−1^, respectively (**Figure** **10**a,b). Such a competitive HER performance is attributed to the increased channel conductance of MoS_2_ nanosheet by adding a positive electric field. Interestingly, the binding energy in the reaction was also found to be sensitive to the electric field. For example, Wu et al.^[^^]^ explored the effects of applied electric field on the electrocatalytic HER performance for the pristine and defective MoS_2_. Both the pristine and the defective systems can thus be activated to a comparable degree by applying the gate voltage (Figure 10c,d). They believed that the added negative surface charge stabilizes *H in all considered cases (Figure 10e). Aside from the thermodynamic properties of catalysis, charge transfer rate and adsorption kinetics also play a vital role in the ideal HER process. For example, Yan et al.^[^ ^279^ ^]^ concentrated on the strategy of applying a back‐gate voltage to the pristine VSe_2_ nanosheet and they revealed the optimization of electrochemical properties is due to the existence of the electric field regulating the adsorption kinetics of the VSe_2_ nanosheets (Figure 10f,g). In addition, many studies have found that the employment of electric field can also affect these aspects of catalysis, such as the contact resistance,^[^ ^177^ ^]^ ionic kinetics,^[^ ^280^, ^281^, ^282^ ^]^ and so on.

Magnetic field also can boost electrochemical reactions. Magnetic effects in electrochemical reactions include magnetothermal effect, magnetohydrodynamics effect, Kelvin force effect, Maxwell stress effect, and spin selectivity effect.^[^ ^283,284^ ^]^ For example, the work of lowering HER overpotential resulted from the magnetothermal effect was proposed by Christiane Niether and co‐workers,^[^ ^277^ ^]^ where the efficiency of HER was improved by FeC–Ni core–shell nanoparticles under a high‐frequency alternating magnetic field. As a result, the HER activity could be generally promoted (Δ*E* = 150 mV at 40.1 mT) in 1 m KOH. Compared to the enhancement of oxygen evolution kinetics equivalent to increasing the cell temperature to 200°C in the OER reaction, the kinetics in the HER reaction only a slight increase, which also provides a reference for the application of magnetic field in HER field. Nevertheless, a different HER reinforcement mechanism holds that the external vertical magnetic field could easily conduct electrons from the glassy carbon electrode to the active site for driving HER, thus achieving magnetic HER enhancement (Figure 10h).^[^ ^285^ ^]^ Unlike the application of magnetic fields in electrochemical processes, applied magnetic fields are also commonly used in the preparation of catalysts.^[^ ^287^, ^288^ ^]^ For example, Wang et al.^[^^]^ proposed a new method to prepare hollow Co_2_P nanoparticles with controllable shell thickness. The application of magnetic field regulates the formation process of Co_2_P hollow nanoparticles, that is, accelerates the dissolution of CoO on the surface of Co nanoparticles and slowing down the Kirkendall effect, thus reducing the particle size and adjusting the shell thickness (Figure 10i).

To better illustrate the advantages of the catalysts prepared under different design strategies, **Table** **2** lists the HER performances of a series of reported electrocatalysts.

**Table 2:** Performance parameters of the discussed electrocatalysts designed for HER

Catalysts	Electrolytes	Activity	Mass loading	TOF	Mass activity	Tafel slope	Main strategies	Refs.
PtCu NSs	0.5 m H_2_SO_4_.	26.8 mV @10 mA cm^−2^	18.9 μg_Pt_ cm^−2^	–	1.08 A mg _Pt_^−1^ @‐0.03 V	28.4 mV dec^−1^	Alloys	^[^ ^83^ ^]^
PtAgCo‐II	0.5 m H_2_SO_4_.	400 mV @705 mA cm^−2^	–	–	–	27 mV dec^−1^	Alloys	^[^ ^87^ ^]^
PtRhCo PAANFs	0.5 m KOH	36 mV @10 mA cm^−2^	0.085 mg cm^−2^	–	2.221 A _Pt_^−1^ 0.5 m EG + 0.5 m KOH	48 mV dec^−1^	Alloys	^[^ ^88^ ^]^
PtNiCo HAMPs	1.0 m KOH	20 mV @10 mA cm^−2^	0.255 mg cm^−2^	–	0.44 mA μg_Pt_^−1^ @‐0.01V	46.3 mV dec^−1^	Alloys	^[^ ^89^ ^]^
H‐PtNiCu‐AAT NPs	0.1 m HClO_4_	32 mV @10 mA cm^−2^	0.1 mg cm^−2^	–	1.2 A mg _Pt_^−1^ @‐0.05V	33 mV dec^−1^	Alloys	^[^ ^90^ ^]^
VMS_2_	0.5 m H_2_SO_4_ 1.0 m KOH	194 mV @10 mA cm^−2^ 206 mV @10 mA cm^−2^	0.5 mg cm^−2^	–	–	59 mV dec^−1^ 89 mV dec^−1^	Compounds	^[^ ^91^ ^]^
Ru‐MoS_2_/CNT	1.0 m KOH	50 mV @10 mA cm^−2^	1 mg cm^−2^	–	–	62 mV dec^−1^	Compounds	^[^ ^92^ ^]^
S‐MoS_2_@C	0.5 m H_2_SO_4_ 1.0 m KOH	136 mV @10 mA cm^−2^ 155 mV @10 mA cm^−2^	1 mg cm^−2^	1.98 s^−1^ @‐0.02 V	–	78 mV dec^−1^ 99 mV dec^−1^	Compounds	^[^ ^94^ ^]^
Fe‐doped NiS_2_	0.5 m H_2_SO_4_	198 mV @10 mA cm^−2^	–	3.39 s^−1^ @‐0.03 V	–	42 mV dec^−1^	Compounds	^[^ ^96^ ^]^
NFP/C‐3	0.5 m H_2_SO_4_ 1.0 m PBS 1.0 m KOH	72 mV @10 mA cm^−2^ 117 mV @10 mA cm^−2^ 95 mV @10 mA cm^−2^	0.4 mg cm^−2^	–	–	54 mV dec^−1^ 70 mV dec^−1^ 72 mV dec^−1^	Compounds	^[^ ^119^ ^]^
Rh_2_P/C NCs	0.5 m H_2_SO_4_	5.4 mV @5 mA cm^−2^	3.7 μg_Rh_ cm^−2^	–	1.72 mA mg_metal_^−1^	–	Compounds	^[^ ^120^ ^]^
w‐Rh_2_P NS/C	0.1 m HClO_4_ 0.5 m PBS 0.1 m KOH	15.8 mV @10 mA cm^−2^ 21.9 mV @10 mA cm^−2^ 18.3 mV @10 mA cm^−2^	12.3 µg cm^−2^	–	–	29.9 mV dec^−1^ 78.4 mV dec^−1^ 61.5 mV dec^−1^	Compounds	^[^ ^121^ ^]^
N‐RuP/NPC	0.5 m H_2_SO_4_ 1.0 m KOH	20.5 mV @10 mA cm^−2^ 29.6 mV @10 mA cm^−2^	0.4 mg cm^−2^	1.56 s^−1^ @‐0.03V 0.72 s^−1^ @‐0.03V	–	31 mV dec^−1^ 35 mV dec^−1^	Compounds	^[^ ^122^ ^]^
Re_3_P_4_@NPVC	0.5 m H_2_SO_4_ 1.0 m PBS 1.0 m KOH	40 mV @10 mA cm^−2^ 70 mV @10 mA cm^−2^ 61 mV @10 mA cm^−2^	0.143 mg cm^−2^	0.34 s^−1^ @‐0.02V	6.20 mA μg_Re_^−1^ @‐0.07V (0.5 m H_2_SO_4_)	38 mV dec^−1^ 77 mV dec^−1^ 62 mV dec^−1^	Compounds	^[^ ^123^ ^]^
IrP_2_@NC	0.5 m H_2_SO_4_ 1.0 m KOH	8 mV @10 mA cm^−2^ 28 mV @10 mA cm^−2^	0.7 mg cm^−2^	–	–	28 mV dec^−1^ 50 mV dec^−1^	Compounds	^[^ ^124^ ^]^
Ni‐GF/VC	0.5 m H_2_SO_4_ 1 m KOH	111 mV @10 mA cm^−2^ 128 mV @10 mA cm^−2^	2.11 mg cm^−2^	0.39 s^−1^ @‐0.15V 0.24 s^−1^ @‐0.15V	–	20 mV dec^−1^ 51 mV dec^−1^	Compounds	^[^ ^133^ ^]^
(Co)‐doped 1T‐MoS_2_	0.5 m H_2_SO_4_	84 mV @10 mA cm^−2^	0.212 mg cm^−2^	–	–	47 mV dec^−1^	Phase transformation	^[^ ^100^ ^]^
Cu@MoS_2_	0.5 m H_2_SO_4_	131 mV @10 mA cm^−2^	0.1 mg cm^−2^	‐240 ± 10 s^−1^ @‐0.02 V	–	51 mV dec^−1^	Phase transformation	^[^ ^102^ ^]^
v‐Pd_2_CoAg NCs	0.5 m H_2_SO_4_	24 mV @10 mA cm^−2^	0.283 mg cm^−2^	–	–	28 mV dec^−1^	Defects	^[^ ^138^ ^]^
Undercoordinated Mo atoms	0.1 m KOH	160 mV @10 mA cm^−2^	–	2.0 s^−1^ @‐0.16 V	–	–	Defects	^[^ ^141^ ^]^
MoS_2_‐60	0.5 m H_2_SO_4_	131 mV @10 mA cm^−2^	–	–	5.43 A g^−1^ @‐0.15 V	48 mV dec^−1^	Defects	^[^ ^143^ ^]^
Dr‐Pt	1 m KOH	26 mV @10 mA cm^−2^	19.5 μg_Pt_ cm^−2^	–	1.16 A mg_Pt_^−1^ @‐0.05V	52 mV dec^−1^	Defects	^[^ ^144^ ^]^
RuRh_2_	0.5 m H_2_SO_4_ 1.0 m PBS 1.0 m KOH	34 mV @10 mA cm^−2^ 12 mV @10 mA cm^−2^ 24 mV @10 mA cm^−2^	0.283 mg cm^−2^	–	1.49 A mg^−1^ @‐0.05V (0.5 m H_2_SO_4)_	17mV dec^−1^ 34 mV dec^−1^ 31 mV dec^−1^	Defects	^[^ ^146^ ^]^
MoS_2_ nanograin	0.5 m H_2_SO_4_	25 mV @onset potential	–	–	–	54 mV dec^−1^	Defects	^[^ ^147^ ^]^
Co@HMNC	1 m KOH	51 mV @10 mA cm^−2^	–	–	–	97 mV dec^−1^	Confinement	^[^ ^148^ ^]^
CoDNG900	0.1 m KOH	193 mV @10 mA cm^−2^	0.25 mg cm^−2^	–	–	90 mV dec^−1^	Confinement	^[^ ^150^ ^]^
Ni_0.25_Cu_0.75_/C	0.5 m H_2_SO_4_	184 mV @10 mA cm^−2^	0.275 mg cm^−2^	8.98 s^−1^	0.64 A mg _Ni_^−1^ @‐0.15 V	84 mV dec^−1^	Confinement	^[^ ^153^ ^]^
Co(10.4)/Se‐MoS_2_‐ NF	0.5 m H_2_SO_4_	104 mV @10 mA cm^−2^	0.5 mg cm^−2^	–	–	67 mV dec^−1^	Confinement	^[^ ^154^ ^]^
NiCo_2_P_2_–ACNTs	0.5 m H_2_SO_4_ 1 m KOH	72 mV @10 mA cm^−2^ 48 mV @10 mA cm^−2^	3 mg cm^−2^	–	–	54 mV dec^−1^ 52 mV dec^−1^	Multisite functionalization	^[^ ^159^ ^]^
Pt/f‐MWCNTs	0.5 m H_2_SO_4_	49.3 mV @10 mA cm^−2^	–	0.921 s^−1^	18.16 A mg_Pt_^−1^ @‐0.05V	30 mV dec^−1^	Multisite functionalization	^[^ ^161^ ^]^
WS_2_@graphene (10 nm)	0.5 m H_2_SO_4_	117 mV @10 mA cm^−2^	0.45 mg cm^−2^	–	–	56 mV dec^−1^	Strain effect	^[^ ^176^ ^]^
D‐IrTe_2_	1.0 m KOH	54 mV @10 mA cm^−2^	0.020 mg cm^−2^	0.71 s^−1^ @‐0.02 V	425 ± 5 A g_Ir_^−1^ @‐0.05V	32.7 mV dec^−1^	Strain effect	^[^ ^177^ ^]^
P‐MoS_2_@HCMF	0.5 m H_2_SO_4_	86 mV @10 mA cm^−2^	0.285 mg cm^−2^	–	–	42.35 mV dec^−1^	Strain effect defects	^[^ ^181^ ^]^
MoS_2_ films on prestretched Ti‐ coated polymer substrates	0.5 m H_2_SO_4_	50 mV @20 mA cm^−2^	–	1.84 s^−1^	–	32 mV dec^−1^	Strain effect	^[^ ^182^ ^]^
Ni*_x_* Mo_1‐_ *_x_* O_2_ (*x* = 0.35)	0.5 m H_2_SO_4_	37 mV @10 mA cm^−2^	–	–	–	37 mV dec^−1^	Doping	^[^ ^199^ ^]^
O,P‐MoS_2_	0.5 m H_2_SO_4_	150 mV @10 mA cm^−2^	102 µg cm^−2^	–	–	53 mV dec^−1^	Doping	^[^ ^203^ ^]^
S‐CoSe_2_	0.5 m H_2_SO_4_	88 mV @10 mA cm^−2^	0.57 mg cm^−2^	–	–	50 mV dec^−1^	Doping	^[^ ^205^ ^]^
MoS_2_/Ti_3_C_2_	0.5 m H_2_SO_4_	280 mV @10 mA cm^−2^	0.36 mg cm^−2^	–	–	68 mV dec^−1^	Promoters	^[^ ^216^ ^]^
MoS_2_/NGDY	0.5 m H_2_SO_4_	186 mV @10 mA cm^−2^	0.286 mg cm^−2^	–	–	63 mV dec^−1^	Promoters	^[^ ^217^ ^]^
2.4% Mn‐doped nanoflower	0.5 m H_2_SO_4_	167 mV @10 mA cm^−2^	0.53 mg cm^−2^	–	–	60 mV dec^−1^	Promoters	^[^ ^218^ ^]^
MoO_2_‐Ni@NC	0.5 m H_2_SO_4_	58 mV @10 mA cm^−2^	–	–	–	35.1 mV dec^−1^	Promoters	^[^ ^219^ ^]^
Pt@NHPCP	0.1 m HClO_4_	57 mV @10 mA cm^−2^	2.0 μg_Pt_ cm^−2^	–	–	27 mV dec^−1^	Nanostructuring	^[^ ^236^ ^]^
Ir‐NR/C	0.5 m H_2_SO_4_ 1.0 m PBS 1.0 m KOH	28 mV@ 10 mA cm^−2^ 86 mV@ 10 mA cm^−2^ 42 mV@ 10 mA cm^−2^	0.283 mg cm^−2^	–	‐0.85 A mg_metal_^−1^ @‐0.01V 3.9 A mg_metal_^−1^ @‐0.01V	23.5 mV dec^−1^ 66.8 mV dec^−1^ 35.2 mV dec^−1^	Nanostructuring	^[^ ^242^ ^]^
NiCo‐nitrides/ NiCo_2_O_4_/GF	1.0 m KOH	71 mV @10 mA cm^−2^	0.5 mg cm^−2^	–	–	35 mV dec^−1^	Nanostructuring	^[^ ^248^ ^]^
CoP/C‐3	0.5 m H_2_SO_4_	–	0.254 mg cm^−2^	–	0.26 mA cm^−2^ @ ‐0.02 V	51 mV dec^−1^	Nanostructuring	^[^ ^249^ ^]^
1T‐MoS_2_/CoS_2_	0.5 m H_2_SO_4_ 1 m KOH	26 mV @10 mA cm^−2^ 71 mV @10 mA cm^−2^	0.42 mg cm^−2^	–	–	43 mV dec^−1^ 60 mV dec^−1^	Interface	^[^ ^9^ ^]^
Mo_2_CT*_x_*/2H‐MoS_2_	0.1 m HClO_4_	119 mV @10 mA cm^−2^	0.75 mg cm^−2^	^–^	0.6 A mg^−1^ @‐0.262 V	60 mV dec^−1^	Interface	^[^ ^270^ ^]^
Ru‐Mo_2_N	0.5 m H_2_SO_4_ 1 m KOH	18 mV @10 mA cm^−2^ 16 mV @10 mA cm^−2^	1.00 mg cm^−2^	–	–	28 mV dec^−1^ 35 mV dec^−1^	Interface	^[^ ^271^ ^]^
Co_9_S_8_‐MoS_2_	0.5 m H_2_SO_4_ 0.1 m PBS 1 m KOH	‐152.1^ ^mV @10 mA cm^−2^ 167 mV @10 mA cm^−2^	526.3 µg cm^−2^	3.10 s^−1^ @‐0.20 V (1 m KOH)	–	‐99.4 mV dec^−1^ 81.7 mV dec^−^1	Interface	^[^ ^272^ ^]^
Mo_2_C/MoSe_2_/Mo	0.5 m H_2_SO_4_ 1 m KOH	80 mV @10 mA cm^−2^ 51 mV @10 mA cm^−2^	–	–	–	49.8 mV dec^−1^ 47.6 mV dec^−1^	Interface	^[^ ^273^ ^]^
RuO_2_/Co_3_O_4_‐RuCo@NC‐1.95	0.5 m H_2_SO_4_	141 mV @10 mA cm^−2^	0.35 mg cm^−2^	–	–	63 mV dec^−1^	Interface	^[^ ^274^ ^]^
SA Co‐D 1T MoS_2_	0.5 m H_2_SO_4_	42 mV @10 mA cm^−2^	0.21 mg cm^−2^	7.82 s^−1^ @‐0.10 V	–	32 mV dec^−1^	Interface	^[^ ^276^ ^]^
Individual MoS_2_@5V	0.5 m H_2_SO_4_	38 mV @100 mA cm^−2^	–	–	–	110 mV dec^−1^	Field effect	^[^ ^277^ ^]^
Pristine MoS_2_@3V Defective MoS_2_@3 V	0.5 m H_2_SO_4_	210 mV @10 mA cm^−2^ 160 ± 5 mV @10 mA cm^−2^	–	–	–	110 mV dec^−1^ 75±1 mV dec^−1^	Field effect	^[^ ^278^ ^]^
VSe_2_ (‐0.8 V)	0.5 m H_2_SO_4_	70 mV @10 mA cm^−2^	–	–	–	59 mV dec^−1^	Field effect	^[^ ^279^ ^]^
ST‐4.65	1 m KOH	131 mV @10 mA cm^−2^	–	–	–	36 mV dec^−1^	Field effect	^[^ ^288^ ^]^

## 4 Applications of HER Electrocatalysts

Hydrogen production from water electrolysis is a good strategy to mitigate the intermittent and fluctuating nature of the most promising renewable energy sources such as light, wave, solar, and wind energy. Currently, electrolytic water splitting technology mainly includes alkaline liquid electrolyzer (AE), proton exchange membrane (PEM), and anion exchange membrane (AEM) electrolyzers. Among them, AE technology has realized industrial‐scale hydrogen production, which is the most mature technology and relatively low‐cost route. However, AE has significant disadvantages such as low energy efficiency and sensitivity to CO_2_.

**Figure 11:**
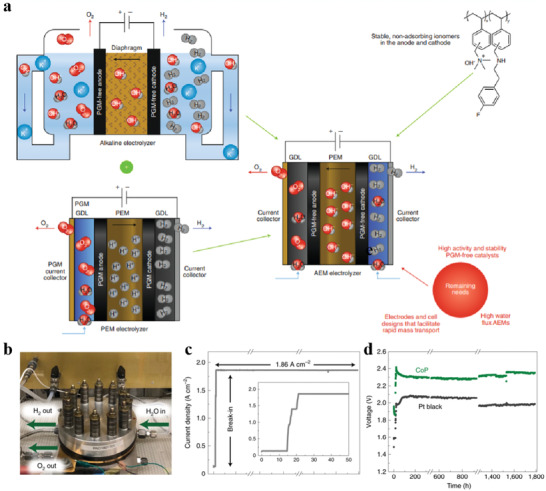
a) Schematic illustration of AE, PEM, and AEM electrolyzers. Reproduced with permission.^[^ ^289^ ^]^ Copyright 2020, Springer Nature. b) Photo of 86 cm^2^ PEM electrolyzer using CoP as HER catalyst. c,d) Durability test. Reproduced with permission.^[^ ^293^ ^]^ Copyright 2019, Springer Nature.

The PEM and AEM electrolyzers consist of a membrane electrode assembly (MEA) and bipolar plates (**Figure** **11**a),^[^ ^289^ ^]^ and the difference lies in the types of ions conducted by the polymer membrane. Compared with AEM, PEM has the advantages of higher current density, higher efficiency, faster dynamic response, and adaptability to the fluctuations of renewable energy generation. Nevertheless, PEM requires the use of expensive precious metal catalyst to accelerate catalysis in corrosive acidic environment, which amounts to an increase in cost. Later, some researchers developed various alternative electrocatalysts and conducted research on HER.^[^ ^290^, ^291^, ^292^ ^]^ For example, King et al.^[^ ^293^ ^]^ demonstrated a low‐cost non‐noble metal CoP catalyst, which has achieved a span from a laboratory scale of 1 cm^2^ to a commercial‐scale PEM electrolyzer of 86 cm^2^ (Figure 11b). Under the same working conditions (400 psi, 50 °C), CoP‐based PEM could continuously produce hydrogen on a 1.86 A cm^−2^ template for more than 1700 h (Figure 11c,d).

The possibility that AEM electrolysis can use non‐noble metal oxides compared to PEM electrolysis represents a significant advantage for AEM over PEM in terms of significantly reducing associated costs.^[^ ^294^ ^]^ However, given the current lack of commercially available AEM, the development of ionic conductive polymers without any aromatic groups to further enhance the transport of water and ions has the potential to drive large‐scale commercial applications of AEM electrolysis.

## 5 Summary and Perspective

Over the past decades, tremendous breakthroughs have been achieved for the design and synthesis of transition metal‐based electrocatalysts as alternatives to Pt‐based materials toward more efficient HER. In this review, we have summarized many descriptors about HER, including Δ*G*_H*_, d band center, and AIE, and so on. These descriptors can be used for rapid screening and predicting HER activity. As a promising strategy for designing high‐performance HER electrocatalysts, alloying, E‐field, and so on have been profoundly discussed in this review. Finally, we also summarized the applications of HER electrocatalysts in PEM and AEM electrolyzers. Nevertheless, there are still several fundamental catalytic constraints that have plagued electrochemical hydrogen production for decades. I) The fundamental HER catalytic mechanism of catalysts under alkaline conditions is still hard to elucidate clearly. For example, some of the classic questions that have plagued alkaline HER for years are “what is the role of OH?” and “whether HBE is a universal descriptor for HER?” II) The short service life of electrode materials results in a decline in catalytic activity. III) The lack of economic and effective noble metal substitutes limits the large‐scale application of electrocatalytic water splitting. More importantly, the measuring environment and indicators of laboratory research are quite different from the actual situation in industrial production, mainly manifested as working parameters, testing system and the working environment of the catalyst and so on. Taking the test system as an example, the best practice in evaluating the mass and/or specific activity of catalysts is to use three‐electrode system with a catalyst loadings of 0.1–0.3 mg cm^−2^ deposited on the conductive rotating electrode substrate. Thus, bottlenecks arising from electrical conductivity and/or mass transport limitation are, at least partially, avoided by applying a rotation. Thus, optimizing MEA for industrial scenarios is a much more complex task than optimizing three‐electrode configuration, and must be carried out for each catalyst and/ or conductive substrate. Hence, the existence of the above factors is tantamount to making the gap between fundamental research and practical industrial application. Therefore, more attentions should be paid to the comprehensive investigation for electrode materials and catalytic mechanisms during hydrogen evolution.

Some future development directions are prospected. I) An in depth, intensive, systematic investigation of the mechanism under alkaline conditions. In depth understanding of the catalyst–intermediates interactions and the local environments, such as local pH and electric double layer is the vitally imperative to unveil slower kinetics with the assistance of innovative experimental methods. Specifically, the structure of the electric double layer is closely associated with the catalytic surface. Besides, such an attempt is likely to be made via getting insight to the catalytic mechanism on the typical nanostructured catalysts, not just traditional single‐crystal Pt alone, that are more reactive and widely used in practice. Likewise, it is of paramount importance to develop more accurate models which describe the relatively complex surface and interface under real HER conditions to narrow the gap between theory and experiments in electrocatalysis. II) Hydrogen production from seawater splitting, avoiding use of the limited freshwater resource, is more intriguing. Toward that goal, the development of seawater splitting could thrive from the following aspects: in‐depth understanding of the reaction mechanism, design better catalysts, and the design and amplification of new electrolytic cells for seawater electrolysis. Thereinto, slow kinetics of seawater electrolysis can be alleviated by replacing oxygen evolution with thermodynamically easier oxidation reactions such as biomass oxidation. III) Considerable efforts should be made to develop low content Pt or Pt‐free catalysts for hydrogen production from water electrolysis. In this regard, the single‐atom catalyst possesses the largest atom utilization efficiency and the unique coordination environment of the active center, which greatly reduces the loading of Pt while maintaining high catalytic activity. IV) Development of highly efficient HER electrocatalyst should meet the high current density and temperature requirements in the industry. Despite considerable efforts to evolve better catalysts, the future development and deployment of water electrolysis at large scale are intrinsically dependent on the constraints associated with the current architecture and design of both PEM electrolyzers and AEM electrolyzers. Similarly, more standardized measurement methods and more reasonable measurement system need to be clarified for lab‐scale evaluation. Especially, a new electrochemical measurement environment simulating the solid–gas interface formed in the MEA is key to assessing local subtle effects in the actual working environment. Overall, we believe that scientists should integrate engineering strategies, not substitute for focusing considerable fundamental researches, when designing better catalysts, membranes and novel architectures and designs of water electrolysis technology, which is essential to translate laboratory findings into large‐scale production. (V) Accurate identification and characterization of catalytic active sites should be given priority in order to understand the catalytic mechanism at the atomic level and design the targeted electrocatalysts. HAADF‐STEM, XAFs, and DFT calculation techniques have proved to be indispensable tools for directly observing catalyst, understanding the configuration and charge transfer process of active center, analyzing coordination model and reaction mechanism, and designing and conceiving novel active center. Meanwhile, the assisted in situ characterization technology can further accurately identify the active sites and deepen the understanding of the basic catalytic mechanism. VI) Emerging technologies, such as artificial intelligence, machine learning, and high‐throughput theoretical computing are also being used to discover new high‐level descriptors, enabling the development of catalyst design strategies. Based on the catalyst design strategies stated above, one can believe the realization of efficient, low‐cost, and sustainable hydrogen energy can be achieved through electrolytic water splitting technology in the future.

## Conflict of Interest

The authors declare no conflict of interest.
